# Evaluation of the applicability of the Cutaneous Lupus Erythematosus Area and Severity Index in Filipino cutaneous lupus erythematosus patients

**DOI:** 10.1016/j.jdin.2026.05.002

**Published:** 2026-05-08

**Authors:** Glen Aldrix R. Anarna, Hanna Lucero-Orillaza, Geraldine T. Zamora, Blessie Marie B. Perez, Victoria P. Werth, Josef Symon S. Concha

**Affiliations:** a Department of Dermatology, University of the Philippines - Philippine General Hospital, Manila, Philippines; b Division of Rheumatology, Department of Medicine, University of the Philippines - Philippine General Hospital, Manila, Philippines; c Department of Dermatology, University of Pennsylvania, Philadelphia, Pennsylvania

**Keywords:** CLASI, CLE, cutaneous lupus erythematosus, Cutaneous Lupus Erythematosus Disease Area and Severity Index, SLE, systemic lupus erythematosus

*To the Editor:* Lupus erythematosus frequently involves the skin, contributing substantially to disease burden and impaired quality of life.^1^ Cutaneous lupus erythematosus (CLE) presents with diverse, sometimes treatment-resistant manifestations that may require systemic therapy.^2^^,^^3^ Accurate assessment is essential for severity stratification, monitoring, and evaluation of treatment response. The Cutaneous Lupus Erythematosus Disease Area and Severity Index (CLASI) is a validated tool designed to measure CLE activity and damage.^4^ However, most validation studies involve predominantly Caucasian populations. Data in Asians, particularly Filipinos with darker and heterogeneous skin phototypes, remain limited. Ethnic and phototype differences may influence lesion morphology and scoring reliability. This study evaluated CLASI’s inter-rater and intra-rater reliability in concordance with the Skin Physician Global Assessment (PGA).^5^

Fourteen adult CLE patients were evaluated by four board-certified physicians (two dermatologists and two rheumatologists). The sample size was statistically powered to estimate 80% reliability with a 10% margin of error, exceeding cohorts in foundational CLASI validation studies. All raters completed standardized CLASI and PGA training prior to independently assessing each patient using both instruments. Inter-rater and intrarater reliability were analyzed using intraclass correlation coefficients (ICCs). Differences in severity classification (mild: 0-9; moderate: 10-20; severe: 21-70) between experienced and newly trained raters were examined using χ^2^ testing. Statistical significance was set at *P* < .05.

Participants were predominantly female (*n* = 13), aged 21 to 61 years with chronic/discoid (*n* = 10), acute (*n* = 3), and subacute (*n* = 1) subtypes. Most had skin phototypes III (*n* = 9) or IV (*n* = 5). To characterize disease distribution, scores were pooled. The overall median CLASI activity was 13.5 (range: 0-30) and damage was 18.5 (range: 2-44). CLASI demonstrated excellent inter-rater reliability for activity (ICC = 0.94) and damage (ICC = 0.87), exceeding PGA reliability (Table I). Intra-rater reliability was excellent for both CLASI and PGA (ICC = 0.97-1.00) (Table II). χ^2^ tests demonstrated that severity classifications were not significantly different between experienced and newly trained physicians for CLASI activity (χ^2^ = 0.11, *P* = .947) or damage (χ^2^ = 0.03, *P* = .984), indicating effective training.

**Table I:** Inter-rater reliability of skin assessment tool scores in Filipino cutaneous lupus patients among dermatologists and rheumatologists

		Agreement ICC	95% CI	Interpretation
All physicians				
Activity	CLASI	0.94	0.84-0.98	Excellent
	PGA	0.84	0.65-0.94	Excellent
Damage	CLASI	0.87	0.68-0.95	Excellent
	PGA	0.70	0.29-0.89	Good
Dermatologists				
Activity	CLASI	0.96	0.87-0.99	Excellent
	PGA	0.76	0.70-0.93	Good
Damage	CLASI	0.93	0.77-0.98	Excellent
	PGA	0.60	−0.02-0.87	Moderate
Rheumatologists				
Activity	CLASI	0.84	−0.04-0.96	Excellent
	PGA	0.77	0.31-0.92	Good
Damage	CLASI	0.84	0.50-0.95	Excellent
	PGA	0.89	0.60-0.97	Excellent

ICC scores: < 0.5 = poor; 0.50-0.70 = moderate; 0.70-0.81 = good; > 0.81 = excellent.

*CI*, Confidence interval; *CLASI*, Cutaneous Lupus Erythematosus Disease Area and Severity Index; *ICC*, intraclass correlation coefficient; *PGA*, Physician Global Assessment.

**Table II:** Intra-rater reliability of skin assessment tool scores in Filipino cutaneous lupus patients among dermatologists and rheumatologists

		Agreement ICC	95% CI	Interpretation
All physicians				
Activity	CLASI	0.97	0.86-0.99	Excellent
	PGA	0.98	0.92-1.00	Excellent
Damage	CLASI	1.00	0.99-1.00	Excellent
	PGA	0.98	0.93-1.00	Excellent
Dermatologists				
Activity	CLASI	0.95	0.49-1.00	Excellent
	PGA	0.99	0.92-1.00	Excellent
Damage	CLASI	1.00	0.97-1.00	Excellent
	PGA	0.99	0.88-1.00	Excellent
Rheumatologists				
Activity	CLASI	0.98	0.81-1.00	Excellent
	PGA	0.98	0.77-1.00	Excellent
Damage	CLASI	1.00	0.97- 1.00	Excellent
	PGA	0.98	0.79-1.00	Excellent

ICC scores: < 0.5 = poor; 0.50-0.70 = moderate; 0.70-0.81 = good; > 0.81 = excellent.

*CI*, Confidence interval; *CLASI*, Cutaneous Lupus Erythematosus Disease Area and Severity Index; *ICC*, intraclass correlation coefficient; *PGA*, Physician Global Assessment.

CLASI demonstrated excellent reliability across specialties and experience levels, outperforming PGA. Reliability remained robust in Filipino patients with darker phototypes, supporting its applicability in Southeast Asian populations. Minimal impact of training on scoring suggests ease of adoption. While activity scores were consistent, damage assessments showed substantial specialty-related variance (between-group variance: 396.44 vs within-group: 76.52), with rheumatologists generally assigning higher scores. This suggests differing subspecialty thresholds for chronic damage, highlighting the need for multidisciplinary calibration in global clinical trials.

Limitations include small sample size and over-representation of chronic CLE. However, this reflects the highly specialized, niche nature of joint rheumatology-dermatology clinics. Potential recall bias during repeat assessments may have influenced intra-rater reliability, although short intervals minimized this risk. Overall, CLASI is a reliable, feasible, and clinically meaningful instrument for assessing CLE in Filipino patients, reinforcing its role as a standardized outcome measure in Southeast Asian research and practice.

## Conflicts of interest

Dr Werth consults for Lilly, Pfizer, Biogen, BMS, Gilead, Amgen, EMD Sorona, CSL Behring, Crisalis, Argenx, Kwoya Kirin, Regeneron, AstraZeneca, AbbVie, GSK, UCB, Rome Pharmaceuticals, Merck, Sanofi, Cabaletta Bio, Nuvig Pharmaceuticals, Takeda Pharmaceuticals, Immunovant, Anaptysbio, Evommune, Innovaderm, Boehringer Ingelheim Pharmaceuticals, Quotient Therapeutics, Duality Biologics, Glycoera, Almirall, Atticus, Inmagene, Exo, Novartis, Xencor, Architect Therapeutics, Celexor, and Bimmunity; received grants from Pfizer, Biogen, Gilead, Corbus Pharmaceuticals, AstraZeneca, Amgen, Regeneron, CSL Behring, BMS, Horizon, Rome Pharmaceuticals, Priovant, Ventus, Viela, Glycoera, and United States Department of Veterans Affairs Merit Review BX005921-01 (Veterans Health Administration, Office of Research and Development and Biomedical Laboratory Research and Development). University of Pennsylvania owns the copyright for the CLASI and shares copyright on the CLA-IGA. Drs Anarna, Orillaza, Zamora, Perez, and Concha have no conflicts of interest to declare.

## References

[1] Patel P., Werth V. (2002). Cutaneous lupus erythematosus: a review. Dermatol Clin.

[2] Werth V.P. (2005). Clinical manifestations of cutaneous lupus erythematosus. Autoimmun Rev.

[3] Okon L.G., Werth V.P. (2013). Cutaneous lupus erythematosus: diagnosis and treatment. Best Pract Res Clin Rheumatol.

[4] Albrecht J., Taylor L., Berlin J.A. (2005). The CLASI (cutaneous lupus erythematosus disease area and severity index): an outcome instrument for cutaneous lupus erythematosus. J Invest Dermatol.

[5] Tiao J., Feng R., Bird S. (2017). The reliability of the Cutaneous Dermatomyositis Disease Area and Severity Index (CDASI) among dermatologists, rheumatologists and neurologists. Br J Dermatol.

